# Structural Features of Nucleoprotein CST/Shelterin Complex Involved in the Telomere Maintenance and Its Association with Disease Mutations

**DOI:** 10.3390/cells9020359

**Published:** 2020-02-04

**Authors:** Mohd. Amir, Parvez Khan, Aarfa Queen, Ravins Dohare, Mohamed F. Alajmi, Afzal Hussain, Asimul Islam, Faizan Ahmad, Md. Imtaiyaz Hassan

**Affiliations:** 1 Centre for Interdisciplinary Research in Basic Sciences, Jamia Millia Islamia, Jamia Nagar, New Delhi 110025, India; mohdamirbiochem@gmail.com (M.A.); parvezynr@gmail.com (P.K.); aarfa30@gmail.com (A.Q.); Ravins@jmi.ac.in (R.D.); aislam@jmi.ac.in (A.I.); fahmad@jmi.ac.in (F.A.); 2 Department of Pharmacognosy College of Pharmacy, King Saud University, P.O. Box 2457, Riyadh, Saudi Arabia; malajmii@ksu.edu.sa (M.F.A.); afzal.hussain.amu@gmail.com (A.H.)

**Keywords:** telomere shortening, mutations, CST complex, shelterin complex, OB-fold proteins, structural genomics, cancer, Telomere replication, small molecule inhibitors

## Abstract

Telomere comprises the ends of eukaryotic linear chromosomes and is composed of G-rich (TTAGGG) tandem repeats which play an important role in maintaining genome stability, premature aging and onsets of many diseases. Majority of the telomere are replicated by conventional DNA replication, and only the last bit of the lagging strand is synthesized by telomerase (a reverse transcriptase). In addition to replication, telomere maintenance is principally carried out by two key complexes known as shelterin (TRF1, TRF2, TIN2, RAP1, POT1, and TPP1) and CST (CDC13/CTC1, STN1, and TEN1). Shelterin protects the telomere from DNA damage response (DDR) and regulates telomere length by telomerase; while, CST govern the extension of telomere by telomerase and C strand fill-in synthesis. We have investigated both structural and biochemical features of shelterin and CST complexes to get a clear understanding of their importance in the telomere maintenance. Further, we have analyzed ~115 clinically important mutations in both of the complexes. Association of such mutations with specific cellular fault unveils the importance of shelterin and CST complexes in the maintenance of genome stability. A possibility of targeting shelterin and CST by small molecule inhibitors is further investigated towards the therapeutic management of associated diseases. Overall, this review provides a possible direction to understand the mechanisms of telomere borne diseases, and their therapeutic intervention.

## 1. Introduction

Naturally, the ends of eukaryotic chromosomes are prone to misrecognized as the double-strand breaks (DSBs) that pose a critical challenge for cell viability and integrity of the genome. The eukaryotic cells overcome this challenge by forming a protective structure at chromosome ends comprising a tandem array of telomeric DNA repeats and telomere-binding proteins [1]. Telomeres of humans are composed of G-rich (TTAGGG) tandem repeats. The duplex telomeric repeat sequences range from 2–14 kb in length in addition to the single-strand overhang of about 12–10 nucleotides [2,3]. The telomere length and its regulations are mainly maintained by three key players namely, telomerase, shelterin, and CST complexes.

**Figure 1:**
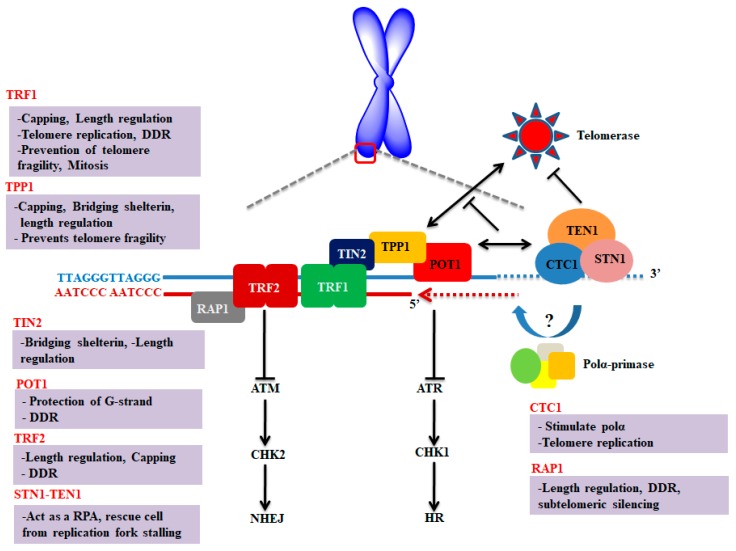
Schematic representation and key molecular function of shelterin and CST complexes at the telomere. The key functions of each component of CST and shelterin complexes were briefly discussed. Shelterin complex proteins are mainly involved in suppressing DNA damage response; however, CST complex is primarily regulating the length of telomere. Figure was adapted and modified from [7,8,9].

The conventional replication mechanism cannot offer the synthesis of chromosomal end which ultimately leads to shortening of telomere at each round of cell division. In normal cells, telomere length is maintained by a ribonucleoprotein (RNP), called telomerase, which is consisting of catalytic (hTERT) and RNA (hTR) components [4,5]. Components of shelterin and CST complexes act as a shield to protect the unique genomic information as illustrated in Figure 1. These two complexes ensure complete replication of the entire genome and thus prevent senescence which is associated with telomere shortening [6,7].

Most of the proteins of these complexes have one or more OB (oligonucleotide/oligosaccharide binding)-fold domains. OB-fold proteins have an affinity to bind nucleic acid and belonging to the nucleic acid-binding superfamily [10,11]. Shelterin consists of six subunits including telomere repeat factor 1 and 2 (TRF1 and 2), repressor/activator protein 1 (RAP1), TRF1-interacting nuclear factor 2 (TIN2), adrenocortical dysplasia homolog (ACD, also referred to as TINT1/PTOP/PIP1 (TPP1) and protection of telomeres 1 (POT1) [6]. Among these six subunits, three components bind with DNA in sequence-specific manner such as POT1 which specifically interacts with single-stranded DNA of telomere (^1^TTAGGGTTAG^10^), whereas TRF1 and TRF2 particularly bound to the double-stranded region of telomeric DNA [12]. In addition, shelterin components perform multiple roles at telomere as they prevent chromosomal ends from being recognized as DNA double-strand breaks (DSB), regulate telomere replication, as well as monitor the telomerase access to telomere [13].

The CST complex is composed of three subunits including, conserved telomere maintenance component 1 (CTC1), suppressor of CDC thirteen homolog (STN1) and telomere length regulation protein TEN1 homolog (TEN1) [14,15,16]. The component of CST (CTC1-STN1) specifically localizes to the single-stranded telomeric DNA and limits telomerase action to prevent overextension of G-overhang. This interaction of CTC1-STN1 to telomeric DNA is stabilized by TEN1 [17]. Moreover, CTC1 originally identified to stimulate DNA polymerase alpha (Polα) [18] and plays a key role in telomere replication [19]. However, the STN1-TEN1 complex of CST acts as a replication protein, A (RPA)-like complex involved in rescuing cells from replication fork stalling during replication stress [20]. In contrast with the shelterin complex which is mainly engaged in the protection of telomere, the CST complex promotes the replication of telomere and does not show any direct role in repressing DDR at telomere [21].

Due to their key role in telomere maintenance, mutations in the CST and shelterin components causes a dramatic alteration in the cellular physiology and often leads to life-threatening diseases including, cancer, bone marrow (BM) failure, dyskeratosis congenital (DC), coats plus (CP), and premature aging syndromes [1,8,14,15,22,23,24]. In this paper, we have extensively analyzed and highlighted the structural features of each component of CST and shelterin complexes, and its importance in maintaining genome integrity. Further, we have analyzed the occurrence of about 115 clinically important mutations based on available literature and broadly discussed their implication in disease development. Finally, we have discussed possible therapeutic opportunities for each component of both complexes in detail. 

## 2. Structure and Function of Shelterin Complex

### 2.1. TRF1 and TRF2

**Figure 2:**
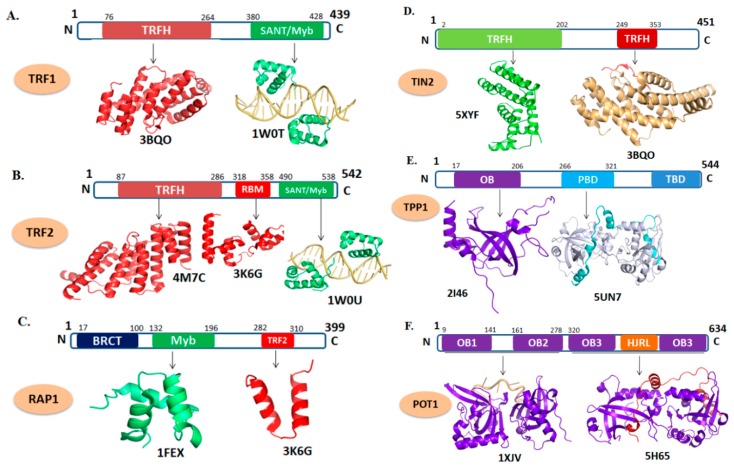
Domain organization and tertiary structure of the shelterin complex. (**A**) telomere repeat factor 1 (TRF1) (PDB ID: 3BQO; depicts the cartoon representation of TRF homology (TRFH) domain of TRF1 in red. 1W0T; the DNA-binding domain of TRF1 shown in green in complex with telomeric DNA which is shown as light orange). (**B**) TRF2 (PDB ID: 4M7C; represents TRFH domain of TRF2. 3K6G; represents the repressor/activator protein 1 (RAP1)–binding motif of TRF2 in red. 1W0U; represents the DNA-binding motif of TRF2). (**C**) RAP1 (PDB ID: 1FEX; represent the Myb domain shown in green and 3K6G; shows RCT domain in red). (**D**) TIN2 (PDB ID: 5XYF; cartoon representation in green depicts TRFH domain and 3BQO; TRF1-interacting nuclear factor 2 (TIN2) peptide shown in red complexed with TRFH domain of TRF1). (**E**) TPP1 (PDB ID: 2I46; depicts the OB-fold domain in purple and 5I2X; represents the POT1 interacting domain of TPP1 (cyan) with C-terminal domain of POT1 (gray)). (**F**) POT1 (PDB ID: 1XJV; depicts the C-terminal domain of POT1 in purple in complex with telomeric DNA (light orange) 5H65; represents the POT1 interacting domain of TPP1 (red) with C-terminal domain of POT1 (purple).

TRF1 and TRF2 are consisting of 439 (UNIPROT-P54274 (TERF1_HUMAN)) and 542 amino acid residues (UNIPROT-Q15554 (TERF2_HUMAN), respectively. Both TRF1 and TRF2 have a similar domain architecture composed of TRF homology (TRFH) at their N-terminal and a SANT/Myb (Swi3, Ada2, N-Cor, and TFIIIB/Myb) DNA-binding domain at C-terminal, which are connected together through flexible hinge region (Figure 2A,B, respectively) [25,26]. The C-terminal SANT/Myb domainof both proteins are almost similar which provide specificity to interact with the double-stranded DNA of telomere [25]. TRF1 and TRF2 interact with DNA as a homodimer or oligomer formed through the interaction of TRFH domain. The TRFH domains are responsible for dimerization and contain a specific peptide docking site for the recruitment of other proteins to the telomere [27]. 

Both TRF1 and TRF2 interact as a homodimer to double-stranded telomeric DNA [28], but they do not interact directly with each other [25]. Interaction of TRF1 and TRF2 to double-stranded telomeric DNA is further stabilized by RAP1 and TIN2. Analysis of the three-dimensional structure of TRF1 and TRF2 reveals that dimer formation is mainly mediated by the respective TRFH domain [29]. The interface of respective TRFH domain contains six α helices (H1, H2, and H9 of each TRFH domain). The H1 and H2 α-helices of one monomer pack against H1 and H2 of another monomer, respectively, to form a symmetrical antiparallel four-helix bundle [29]. Despite a high structural similarity in TRFH domains of both proteins, they do not interact with each other mainly due to incompatible hydrophobic networks that form the dimerization interface. The amino acid residues involved in intermolecular interactions of TRFH domains of both TRF proteins play an important role in maintaining the dimer interface [29]. Mutations of amino acid residues of the TRFH domain (dimer interface) effects protein-protein and protein-DNA activities.

Inactivation of the *TRF1* gene in the mouse embryonic fibroblasts (MEFs) resulted in the stalling of DNA-replication process at the telomere which leads to the formation of gaps, called fragile telomeres [30,31]. Inactivation of the *TRF1* gene and its subsequent abortive replication enhances the activation of robust DNA damage response (DDR) [32,33]. However, inactivation of the *TRF2* gene resulted in the telomere fusion mediated by NHEJ events independently of replication fork stalling [34]. Up-regulation or down-regulation of *TRF1* and *TRF2* genes has been reported in many diseases including cancer [35,36,37]. 

### 2.2. RAP1

RAP1 consists of 399 amino acid residues (Uniprot ID-Q9NYB0), and has three distinct domains (Figure 2C). The N-terminal BRCT (BRCA1 C-terminus) domain is responsible for the recognition of phosphorylated peptide. The C-terminal TRF2 domain interacts with TRF2 protein. The Myb domain generally used to binds with telomeric DNA in budding yeast that has two copies of Myb domain. However, the mammalian RAP1 has only one Myb domain and do not involve in such interaction and therefore RAP1 dependent on TRF2 for telomeric interaction [38,39]. The architecture and topology of mammalian Myb domains are very close to that of budding yeast and TRF1; however, the surface electrostatic potential of the mammalian Myb domain is distinct from that of other Myb domains. Myb domains that have DNA-binding activity exhibit a positively charged large surface closely with the highly negatively charged backbone of DNA. Conversely, mammalian Myb domain shows no distinct positive surface, reveals its lack of DNA-binding property [40].

RAP1 interacts with telomeric DNA only through TRF2 which is essential in chromosome ends protection since its deletion from telomeres resulted in ATM-dependent DNA damage signaling and NHEJ pathway-mediated massive end-to-end fusions [41]. Inactivation of RAP1 resulted in the telomere shortening, hyperpigmentation, and enhanced DDR activation [42]. Recently, two independent studies from different groups demonstrated that mutations in the *RAP1* gene are associated with chronic lymphocytic leukemia and familial melanoma [43,44]. 

### 2.3. TIN2

TIN2 protein is consists of 451 amino acids (UNIPROT-Q9BSI4 (TINF2_HUMAN)) and comprised of two distinct domains (Figure 2D). TIN2 interacts with TPP1 and TRF2 through two different interacting modules [27], one is the N-terminal domain (residues 2–202), which recognized a short TIN2-binding motif of TRF2 (residues 350–366). The other is the short TRFH-binding motif (TBM) (residues 256–276) at the C-terminal portion of TIN2, which interacts with TRFH domains of both TRF1 and TRF2 [27]. Interactions of TIN2 with these proteins develop a bridge between the components of shelterin complex and ssDNA or dsDNA of telomere [45,46]. In the ternary complex, each polypeptide of the TIN2-binding motif of TPP1 is folded into a helix–loop–helix motif. Both helices and the connecting loop make extensive contacts with TIN2 (residues 2–202). The driving force for the binding of TIN2-binding motif of TPP1 to TIN2 (residues 2–202) is van der Waals interactions, as most conserved residues of TIN2-binding motif of TPP1 are hydrophobic in nature. The core of this extended interface between TIN2-binding motif of TPP1 and TIN2 (residues 2–202) consists of a panel of hydrophobic residues from both proteins. The extensive contacts among the side chains of these residues mediate the specificity of TIN2-binding motif of TPP1 recognition by TIN2 (residues 2–202).

TIN2 act as an adaptor which plays crucial role in stabilizing the subunit of shelterin complex. It binds both TRF1 and TRF2, providing stability to these proteins at telomere [46]. In addition, it is also interacting with TPP1 and is indispensable for the recruitment of TPP1/POT1’s to the shelterin complex. As the central hub of the shelterin complex, TIN2 plays pivotal roles in telomere maintenance and end protection [46,47,48,49,50,51]. Inactivation or loss of TIN2 results in the activation of DDR, proliferative arrest, CHK1, and CHK2 phosphorylation. Thus, activation of ATM and ATR pathways take place at telomeres deficient of TIN2, and this deficiency results in the commencement of repair mechanisms which in turn activates the alternative lengthening of telomere (ALT) pathway. On the other hand, knockdown studies of the *TIN2* gene in model organisms show a reduction in the TRF2 function, telomerase recruitment to the telomere [52,53], abnormal mitochondrial morphology, reduced glycolysis and increase oxidative metabolism [52]. Furthermore, the expression of a mutant form of TIN2 or deletion imposes a severe effect on the stability of the shelterin complex [46,54]. Till date, more than 25 clinically important mutations are reported which are known to alter the cell survival and ultimately resulted in DC and pulmonary fibrosis [55,56].

### 2.4. TPP1

TPP1 is encoded by *ACD* gene and consisting of 544 amino acids (UNIPROT ID-Q96AP0 (ACD_HUMAN)). TPP1 is a multidomain protein with confined regions that act as interacting sites for other shelterin proteins and telomerase [57,58,59,60]. The N-terminal of TPP1 comprises an OB-fold (amino acid residues, 87–250) domain, a centrally positioned POT1 binding domain (PBD) (residues, 250–334), and a C-terminal TIN2 binding domain (TBD) (residues 510–544) were also reported (Figure 2E). The structural analysis of the OB-fold domain reveals a typical OB-fold architecture that is common among many DNA-binding proteins [57]. OB-fold domains have been implicated in coordinating protein–protein interactions within multi-component complexes [61,62]. In this regard, the OB-fold domain of TPP1 helps in the establishment of interactions with the telomerase facilitating its recruitment to the telomere [53,58,63,64]. In addition, the OB-fold domain of TPP1 interacts with the TERT component of telomerase and involved in regulation or recruitment of telomerase to the telomere [46,65]. The TEL (TPP1’s glutamate (E) and leucine-rich (L)) patch of this domain is responsible for telomerase recruitment at telomere [58,64,66]. Several mutations in TPP1 gene have been found that are coupled with many diseases including cancer [43]. 

### 2.5. POT1 

POT1 is consisting of 634 amino acid residues (UNIPROT-Q9NUX5 (POTE1_HUMAN)). It has four distinct domains, including two N-terminal OB-fold (OB1 and OB2) domains and a third OB-fold (OB3) along with Holiday junction resolvase (HJR) domains at C-terminal of POT1 (Figure 2F) [67]. POT1 and telomeric DNA interaction are mediated by the two OB-fold domains of POT1, while the C-terminal region binds with TPP1 [65,68]. POT1 binding to TPP1 enhances its DNA binding properties by 10-fold [57,60,69]. The POT1–TPP1 complex recruits telomerase to the ends of the chromosome [57,65] through direct contacts of telomerase with the TEL patch situated at the N-terminal OB-fold of TPP1 [58]. In addition, POT1 interaction to the telomeric DNA overhang helps in resolving G-quadruplexes and allows telomerase loading to telomeres for telomere extension [70]. 

The N-terminal of POT1 adopts an elongated conformation and is composed of two OB-fold domains closely connected by a short linker [71,72]. The ssDNA of telomere in the complex spans both OB1 and OB2, binding in the continuous concave groove [73]. Further, it is reported that OB1 of POT1 makes much more extensive contact with the ssDNA than OB2 [22,73].

The C-terminal of POT1 consists of 330–634 amino acid residues which interact with the central OB-fold domain of TPP1 (255–337 amino acid residues). The C-terminal of POT1 consists of an OB-fold and a HJR domain. The canonical OB-fold of C-terminus is structurally like Telomere End-Binding Protein α (TEBPα, PDB ID: 1OTC) of *Oxytricha Nova.* The TPP1 helix α1 is leucine/valine rich and the majority of contacts with the POT1C, HJR are hydrophobic in nature [67].

POT1 is essential for the inhibition of DDR-mediated ATR pathway and implicated in the regulation of 3′ G-strand overhang [74]. By its strong affinity to the 3′ overhang at telomere, POT1 acts as a natural inhibitor of telomerase. Any alteration in the DNA-binding domain of POT1 is leading to excessive telomere elongation, signifying unregulated access of telomerase to the telomere [75]. Silencing of either *POT1* or *TPP1* gene through the RNA-interference (RNAi) technology increases the telomere length and chromosomal instability [76], indicating its role in the maintenance of telomerase access to the telomere overhang [73]. Since the POT1 plays a key role in the protection of telomere concerning telomere elongation and cell immortality, it has been considered as a promising drug target for cancer therapy [77]. Several mutations in the *POT1* gene have been identified in different types of melanoma and CP patients [78]. 

## 3. Structure and Function of CST Complex

### 3.1. CTC1 

**Figure 3:**
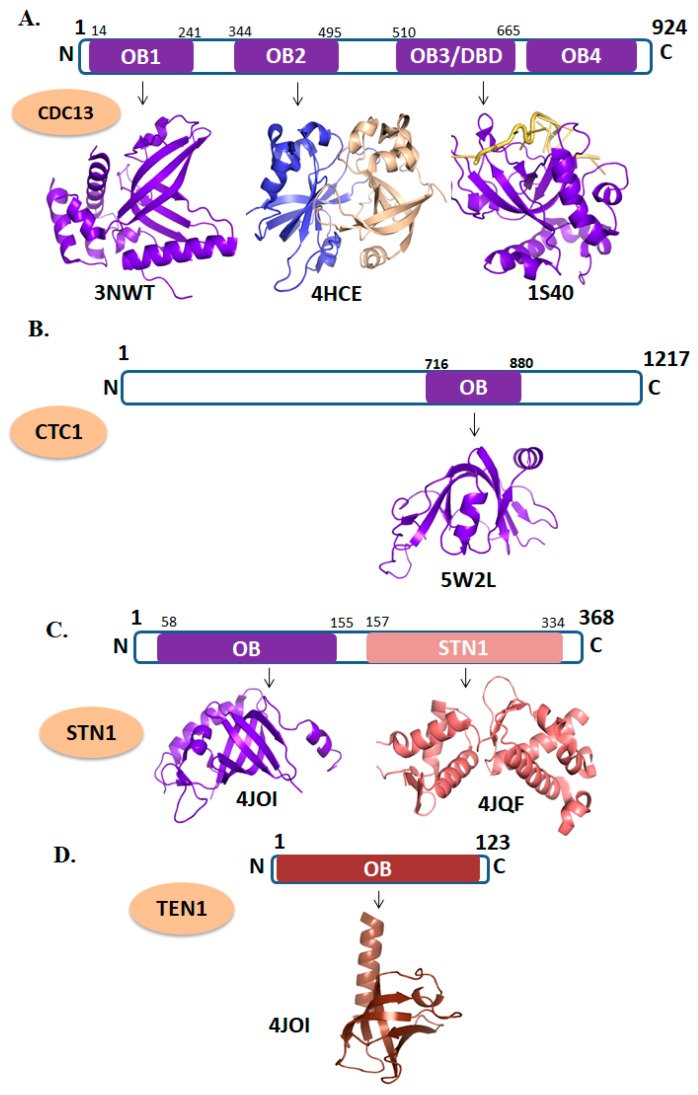
Domain organizations and tertiary structures of CST (CTC1-STN1) complex. (**A**) CDC13 (PDB ID: 3NWT; OB1 domain shown in purple, 4HCE; represents the dimer of OB2 domain, 1S40; depicts the DNA-binding domain of CDC13 in complex with telomeric DNA). (**B**) CTC1 (PDB ID: 5W2L; represent the OB-fold domain of CTC1 in purple. (**C**) STN1 (PDB ID: 4JOI; cartoon representation of N-terminal OB-fold domain of STN1and 4JQF depicts the N-terminal domain of STN1 in salmon color). (**D**) TEN1 (PDB ID: 4JOI; depicts the OB-fold domain of TEN1 in chocolate color).

CTC1 is composed of 1217 amino acid residues (UniProtKB-Q2NKJ3 (CTC1_HUMAN)). The CDC13 is a yeast homolog of mammalian CTC1 which has four OB-folds domains (Figure 3A). Recently, a high-resolution structure of an OB-fold domain of CTC1 has been solved which is found at the C-terminal region of the protein (Figure 3B). In mice, deletion of the *CTC1* gene resulted in a rapid loss of C-strand telomeric DNA which causes catastrophic telomere loss and premature death [21]. Taken together, it has been proposing that the CTC1 is vital for telomere length maintenance by promoting efficient telomere replication and C-strand protection. As CTC1 have critical role in CST complex formation its mutation brings lethality in the cellular environment [79]. Several naturally occurring mutations are known in CTC1 which are associated with CP and DC [80,81]. 

### 3.2. STN1-TEN1

The STN1 (UniProt KB-Q9H668) and TEN1 (UniProt KB-Q86WV5) are comprised of 368 and 123 amino acids residues, respectively. STN1 is consist of two domains, the N-terminal OB-fold and C-terminal STN1domain (Figure 3B) [15], whereas, TEN1 is composed of a single OB-fold domain (Figure 3C). Human TEN1 does not interact with ssDNA of telomere which is in contrast to the yeast TEN1 [16,82]. Conversely, human STN1 binds with ssDNA with high affinity but lacking specificity [82]. High affinity and specificity of the human CST complex for single-stranded telomeric DNA are provided by the larger component of CST, CDC13/CTC1 [83]. Furthermore, STN1-TEN1 complex formation is vital for the proper functioning of CST complex. Mutations in the *STN1* gene have been associated with CP [15,84] in contrast to the mutations in the *TEN1* gene [16,82].

## 4. Diseases

Several human diseases are associated with the telomere shortening including, liver cirrhosis, ulcerative colitis, atherosclerosis, cardiovascular disease, cancer and many premature aging syndromes [85,86,87,88]. Here, we have extensively analyzed the mutations in CST and shelterin complexes and their association with disease development. Some clinically important mutations, associated genes, and their corresponding cellular fitness are shown in the Table 1. 

### 4.1. Role of Shelterin Complex in Cancer

The structures of telomeric DNA is comprised of tandem repeats of G-rich (TTAGGG) sequences protected with shelterin complex. Shelterin complex is comprised of six proteins (TRF1, TRF2, TIN2, POT1, TPP1, and RAP1) which is essential for telomere protection, chromosomal stability and regulation of telomere length. Further, the shelterin complex implicated in the modulation of telomerase activity at chromosome ends recognizes telomeric DNA and remodels it into a t-loop. This process shelters the 3′ overhang from being recognized as DNA damage. Changes in the structure and function of any of the components of this complex may lead to undesirable DDR that often leads to the development of tumorigenesis and cancer progression [110,111,112].

#### 4.1.1. RAP1

Several mutations are reported in all three domains of RAP1 which are associated with cancer. Some of the critically important mutations are, p.M5I, p.D10H, p.Q191R, and p.R364X, found in the patient of familial melanoma, whereas mutations including p.A104P, p.R133Q are reported in the CLL [43,44]. In addition, a nonsense mutation p.R364X in the *RAP1* gene leading to the formation of a C-terminal truncated protein that disrupts the TRF2-binding domain, and subsequently diminishes the binding to shelterin complex, which possibly leads to melanoma susceptibility [43]. Additionally, p.Q191R and p.M5I mutations are noticed in the patients of cutaneous malignant melanoma (CMM), sporadic melanoma, and ovarian cancer. These mutations are found in the Myb and BRCT domains, respectively, and considerably affect the interaction of RAP1 to telomeric overhang. Due to this, RAP1 is unable to regulate telomere homeostasis and disturb cellular fitness [43]. Recently, two novel mutations (p.A104P and p.R133Q) were also reported in CLL patients [44]. 

#### 4.1.2. TPP1

Interaction of shelterin complex and hTERT has been carried out by TPP1 and POT1 complex [107]. Mutations or inhibition of the TPP1/POT1 subunit increases the telomere length, which clearly shows that TPP1/POT1 subunit is essentially required to inhibit the elongation of telomere [64,67]. A nonsense mutation p.Q320X in the *TPP1* gene was recently identified which disrupts the POT1 and TIN2 binding, which ultimately resulted in the formation of a non-functional shelterin complex [113]. In consistence, mutations such as p.V272M and p.N249S were identified in the lower-density melanomas, whereas p.A200T and p.I322F mutations were observed in the CMM patients. Among the five mutations discussed above, four mutations were found in the POT1-binding domain of TPP1 that is critical for the proper functioning of the shelterin complex. More recently, a novel mutation, p.G223V was also identified next to the TEL patch in the OB-fold domain of TPP1 that interacts directly with the catalytic subunit of telomerase. TPP1, as a subunit of shelterin complex was particularly shown to binds with POT1 to shield telomeres and recruit telomerase to the chromosome ends [58,63,64]. Further extensive analysis of p.G223V mutants shows an increase in the cell survival of childhood pre-B acute lymphoblastic leukemia (cALL), and protect leukemia cell from apoptosis and increase the telomere length [94]. Further studies are needed to translate the underlying molecular mechanisms implicating TPP1 in apoptosis inhibition of leukemia cells. Altogether, these data strongly support a role for TPP1 p.G223V mutant in promoting leukemia cell maintenance. Interestingly, recurrent somatic mutations in the OB-fold domains of POT1 reported causing telomere dysfunction in CLL signifying that alteration of TPP1-telomere binding could lead to genomic instability and cancer [114].

#### 4.1.3. POT1

POT1 was the first identified member of the shelterin complex which is mutated in cancer. Mutation in POT1 mainly found in OB-fold domain which plays critical role in telomeric DNA binding [101]. POT1 mutations are not confined to the OB-fold domain only, instead it spread through entire length of POT1 [78,97,101,102], and likely causative of many disease including CLL [101,102], familial melanoma [96], CMM [97], CP [100], cardiac angiosarcoma [99], and familial glioma [98]. Most of the mutation is clustered in the OB-fold domain which disrupts the POT1′s ability to interact with telomeric DNA and does not able to regulate the length of the telomere. Patients carrying these mutations have a very high probability of sister chromatid fusions or sometimes chromosome fusion or may have fragile chromosomal ends [8,101]. 

In two independent studies, researchers found many significant mutations in the OB-fold region of the *POT1* gene which are coupled with CLL [101,102]. Cells from CLL patients have telomeric and chromosomal abnormalities which indicated that mutations in *POT1* gene support the acquisition of the malignant features. Due to the discovery of *POT1* as a commonly mutated gene in CLL which perhaps facilitates novel approaches for the clinical management of this disease [101,102]. Currently, three more mutations (p.Y36C, p.Q376R, p.Q358S) in the *POT1* gene have been identified from CLL patients, predicted to disrupt the interaction of POT1 with TPP1 and telomere overhang which consequently contributed to the CLL pathogenesis [44].

Robles-Espinoza and co-worker [96] identified p.Y89C, p.Q94E and p.R273L mutations in the *POT1* gene associated with familial melanoma. Most of the mutations either effects the splicing of POT1 mRNA or alter key residues in OB-fold domains [96]. These mutations disrupted the protein-telomere interaction and lead to telomere lengthening [96]. In consistence, three novel mutations (p.G95C, p.E450X, p.D617Efs) have been identified in the *POT1* gene from glioma patients [115,116,117]. Out of three, two mutations in the *POT1* gene (p.G95C, p.E450X) are predicted to destabilize the POT1 structure, thus shelterin complex. Mutation in this region affects the DNA-binding as well as TPP1 binding property of POT1 [98]. Along this line, p.D617Efs mutation is predicted to disrupt the TPP1-binding [98]. Moreover, p.R117C in the *POT1* gene is associated with cardiac angiosarcoma (CAS), a rare malignant tumor, whose genetic basis is not fully understood [99].

### 4.2. Role of Shelterin Complex in DC and CP

Recently, several reports highlighted the importance of shelterin complex in hematopoiesis, DC and CP [8,118]. DC is characterized by a disordered human telomere with many pleiotropic manifestations that often lead to the BM failure [119,120]. However, CP is a rare autosomal disorder with intracellular calcification [100]. In addition to the mutations in shelterin complex genes, a large number of mutations in other genes are also reported as a major cause for DC [121,122]. Here, we briefly discuss only the shelterin complex genes implicated in DC and CP.

#### 4.2.1. TIN2

TIN2 was the first identified component of shelterin, implicated in DC. Patients harboring mutation in the *TIN2* coding gene usually has a shorter telomere with symptoms appear at an early age [92,123]. Mutation in this gene is mainly associated with variants of DC called Hoyeraal-Hreidarsson (HH) [124] and Revesz syndromes [92,125]. Currently, more than 25 mutations are reported from DC patients and its variants [89,90,91,92]. All mutations reported are clustered in the exon 6a, corresponding to the amino acid 269–298 [91,92,125,126]. Domain organization of TIN2 shows that all of the mutations occurs in the TRFH domain which is responsible for the interaction of TIN2 with TRF1 [27]. Shortening of telomere occurring due to TIN2 mutations has been explained by two mechanisms that may affect the maintenance of telomere length [51,127]. These mechanisms are based on the reduction in recruitment of telomerase in TPP1-dependent where telomere shortening was not accompanied by changes in total telomerase activity, TIN2 localization, or telomere end protection. Interestingly, TIN2 participates in the TPP1-dependent recruitment of telomerase activity and compromise the telomere recruitment of telomerase, leading to telomere shortening and the associated pathogenesis [127]. However, another report shows that telomere shortening also occur through TPP1-independent manners, where they indicate that TIN2-R282H mutation elongates telomeres at a reduced frequency as this mutation affects the telomerase-telomere co-localization, separable from its role in telomere protection [51]. In addition, studies on the p.K280E mutation are associated with telomere shortening and DDR activation [91,128]. In addition, a study has also shown that DC-linked mutation disrupts the binding of TIN2 with HP1γ (a heterochromatin protein) which affects the cohesion of normal sister telomere [129]. 

#### 4.2.2. TPP1

Mutation in the *TPP1* gene is found to be associated with the inherited BM failure and DC [95,130]. p.K170X mutation is associated with telomere shortening as this mutation brings structural changes in TEL patches (a small region in OB-fold of TPP1). Structural changes in TEL patches inhibit the TPP1’s ability to bind telomerase and finally results in the DC pathogenesis. While, mutation substituting threonine for proline at position p.T491P in the TIN2-interacting domain of TPP1 does not affect the recruitment of telomerase but resulted in modest disruption of the TPP1/TIN2 interaction, the consequence of which still unknown [130]. 

In consistence, p.K170X mutation in DC causes cancer progression [130]. These findings suggest that TEL patches of TPP1’s are important in maintaining telomere homeostasis. In addition, Zhong and co-worker [64] generated several mutations (single and double mutant) in TEL patch in order to see the effect of these mutations on efficacy of TPP1’s to recruit telomerase to the telomere. They identified a putative interaction surface that monitors the interaction between telomerase and TPP1 and hence mutation in TEL patch is no longer able to regulate the telomere homeostasis [64].

Mutation in TEL patch causes defective recruitment of telomerase to telomeric ends and this defect is supposed to result in telomere shortening as observed in affected individuals [95,130]. These findings supported the study of engineered mutations in embryonic stem cells [131]. Interestingly, mutations in the TEL patch have not been translated in vivo, suggesting the pathogenic mechanism remains to be fully investigated. 

#### 4.2.3. POT1

Apart from a reported *POT1* gene mutation in cancer, its p.S322L mutation causes CP. Mutation in the *POT1* gene probably disrupts the POT1/CST-dependent telomere fill-in and this deficiency in the fill-in step generates truncated telomeres that halt the proliferation of cells lacking telomerase [100].

### 4.3. CST Complex and Diseases

#### 4.3.1. Role of CTC1 in CP and DC

There are several naturally occurring mutations in *CTC1* gene that resulted in a range of rare genetic disorders such as CP and DC. Several patients with CP have shortened telomeres, suggesting that telomere dysfunction/telomere shortening possibly plays an important role in CP disease development. Mutations in *CTC1* gene are responsible for CP spread throughout the genome, and their associations with human disease are typically biallelic [7,104,105]. Approximately 20 mutations have been reported in the *CTC1* gene which either abrogates the interaction of CTC1 with ssDNA of telomere or DNA polα or to the STN1-TEN1 subunit of CST complex [104,132]. 

Interestingly, three naturally occurring mutations at the N-terminal and central region of CTC1 (p.A227V, p.V259M, and p.V665G) interrupt CTC1/polα-primase binding to the telomere. Particularly, the p.V259M mutation resulted in a significant accumulation of telomere-free ends. Furthermore, mutation including p.L1142H, p.A227V, p.V259M, p.R987, and p.1196-Δ7 (deletion of amino acid residues 1196–1202) not only interrupt the association of CTC1-STN1 and polα-primase to telomere but also negatively affect the nuclear localization of this complex [7,104,105,106,133]. In addition, there are four novel mutations (p.C424G, p.L247P, p.L464V) in *CTC1* gene have been identified in DC [134]. The lethality of these mutations were identified using bioinformatics tool called PolyPhen2, which shows that these mutation are probably lethal [134].

Few novel mutations in the *CTC1* gene (p.H484P, p.G278V, p.Y281H) have been identified in Indian families. These mutations were clustered in the N-terminal OB-fold domain of CTC1 which might induce conformational changes in protein structure that ultimately effects the reduction in binding affinity of CTC1 with ssDNA. This change in binding affinity compromises the telomere structural integrity which ultimately leads to CP [106,107]. 

#### 4.3.2. STN1-TEN1

STN1 participates in multiple aspects of telomere homeostasis and mutation in this gene is coupled with CP and DC [84,109,135]. Recently, C. Bryan and co-worker [82] have generated single and double mutants (p.D78A, p.I164A, and p.R27Q, p.Y115A, p.R119Q) in *STN1* and *TEN1* gene, respectively to determine the effects of these mutations on STN1-TEN1 complex formation [82]. They further reported that single mutants showed a moderate loss of binding affinity while double mutants abolished STN1-TEN1 binding. Loss of binding affinity between STN1 and TEN1 occurs due to the disruption of a key salt bridge and hydrophobic interactions between these two CST subunits. This disruption in STN1-TEN1 complex formation results in a non-functional CST complex which leads the formation of elongated telomere and chromosomal abnormalities [82]. 

A mutational study on the OB-fold domain of STN1 was performed to address the effect of mutations on DNA-binding properties of STN1. Bhattacharjee and co-workers [109] generated three mutations (p.W89A, p.R139L, p.Y141A) where p.W89A and p.Y141A mutations were chosen because of the equivalent mutations in mouse STN1 decrease DNA-binding approximately 60% in pull-down assays [135] which is well translated through in vivo experiments [109]. However, from in vitro studies, they found that STN1 mutation disrupts binding to short DNA substrates only [109], suggesting that CST actually binds to DNA in a dynamic fashion which could provide a mechanistic explanation for how CST helps to resolve a diverse array of replication problems to preserve genome stability [109]. Two novel mutations (p.R135T, p.D157Y) are reported in the *STN1* gene and these mutations were found to be pathogenic in nature [84].

## 5. Therapeutic Strategies against Telomere Maintaining Components

**Figure 4:**
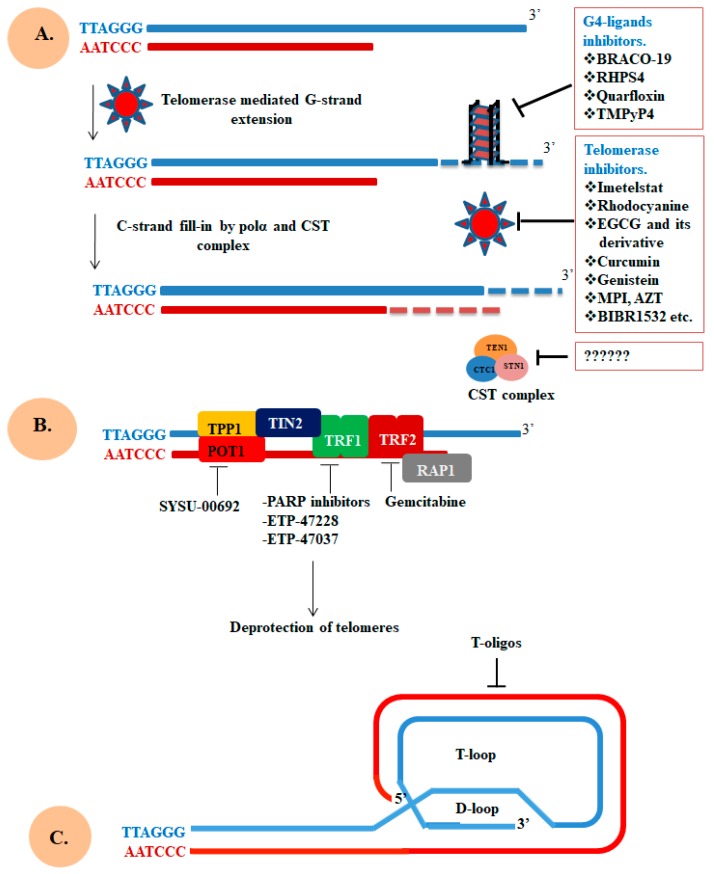
Molecular target directly affecting the integrity of telomere. (**A**) G4-ligands bind with telomeric end to stabilize or promote the formation of G4 structure which abrogates the telomeric end extension. Telomerase inhibitors target either hTERT or hTR and block telomere extension, consequently disrupting telomere integrity. The CST complex is crucial for C-strand synthesis and defect in this complex dysregulate telomerase action and telomere loss. (**B**) Targeting components of the shelterin complex involved in the protection of telomeric end. (**C**) Use of T-oligos technology to block the lengthening of telomere. Figure is adapted and modified from the papers [192,206,207].

**Figure 5:**
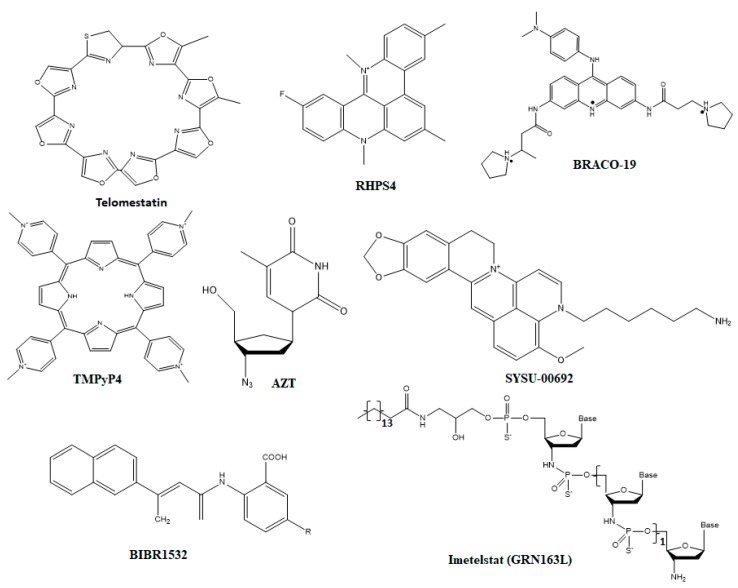
Structures of small molecule inhibitors that target different molecular interactions responsible for the telomere integrity. The chemical structures compounds depicted here have binding affinity against protection of telomeres 1 (POT1).

Here we try to highlight the telomerase, telomere, CST, and shelterin complexes as a therapeutic target to address a large number of diseases associated with telomere malfunctions. Telomere instability fails to maintain the integrity of the genome and often coupled with a shortening of telomere and its fusion [31,136]. Instability of telomere occurs due to several reasons, one of the well-known is the shortening of telomere with each cell division [137]. Furthermore, instability of telomere can arise when telomeric DNA form G-quadruplexes (G4) which interferes with the synthesis of telomeric DNA and result in telomere fragility and possibly rapid loss of telomere [138]. In addition to telomere shortening and formation of G4 structure, telomere instability can also results from the deprotection of telomere which is enhanced due to unavailability of telomere binding proteins, and this unavailability leads to the loss of DDR suppression and enhancement of genomic rearrangements [139,140]. Since, the stability of telomere contributes to the replication immortality in cancer cells, thus targeting telomere stability through interfering with the telomere synthesis carried out by telomerase or protection may provide a newer approach for the treatment of cancer and other telomere diseases. Key molecular targets, which play a direct role in maintaining telomere integrity, are shown in Figure 4. Some important small molecules inhibitors which inhibit the function of these molecular targets are shown in Figure 5. Details of these inhibitors and their mechanism of action are listed in Table 2.

### 5.1. Telomerase

Therapeutic targeting of telomerase is a continuously growing and evolving approach to treat telomere borne diseases, as telomerase overexpression is associated with almost all types of cancer [184,185]. As we discussed earlier, human telomerase is consisting of two core components: the hTERT and hTR. Presently, there are many classes of compounds tested that either target hTERT or hTR for their ability to suppress or inhibit the growth of tumor [186,187,188]. A large number of small molecules including those screened from chemical library of reverse transcriptase show direct interaction with hTERT. Along this line, a compound (BIBR1532) binds directly to the hTERT and inhibits the catalytic function of telomerase [141,146], promote shortening of telomere and senescence in human cancer cells [142]. However, further studies show that BIBR1532 could be promising when given in combination with traditional chemotherapeutic agents [144,145]. 

Other effective inhibitors such as isothiazolone (TMPI) [148], epicatechin (EGCG and MST312) [149,150] and derivative of bisindole are reported to be selective against telomerase. Also, quinoxaline [189], nitrostyrene [190], rubromycin analogs [191], chrolactomycin, and helenalin have been demonstrated to block the action of telomerase [192,193]. 

Moreover, other molecules including inhibitors of cyclooxygenase-2 (COX-2) [194], histone deacetylase (HDAC) inhibitors [195,196], and tyrosine kinase inhibitors [197] show inhibitory effect on telomerase along with several other key physiological processes [198,199]. Together with these compounds, other molecules that specifically inhibit phosphorylation of telomerase such as bis-indolylmaleimide I and H-7,231 antioxidants (vitamin E) [200], anti-inflammatory nonsteroidal drugs (aspirin) are reported to have inhibitory effects on telomerase [192]. In consistence, several other nucleoside analogs are considered as promising drugs for the inhibition of telomerase [201,202]. These inhibitors include azidothymidine (AZT) [151], l-enantiomers (L-dTTP and L-dGTP) [203], 6-thio-2′-deoxyguanosine 5′-triphosphate (TDG-TP) [204]. Carbovir 5′-triphosphate, 2′,3′-Dideoxyguanosine 5′-triphosphate and D-carbocyclic-2′-deoxyguanosine 5′-triphosphate have an inhibitory effect on the telomerase [205].

In addition to targeting hTERT, some novel chemical approaches has been implemented to target the RNA component of telomerase (hTR) while giving a special emphasis on the 11-mer template region (5′-CUAACCCUAAC-3′). Such molecules are called small interfering RNA (siRNA) or ribozymes, one such oligonucleotide known as Imetelstat (GRN163L) inhibit hTR to forming an active complex with hTERT, and hence interferes with telomerase activity, promote telomere shortening, senescence, or apoptosis [208]. Interestingly, observation from several clinical trials support for the potential use of Imetelstat to treat patients harboring hematological cancers and also for those which have solid lung tumors with short telomere [209]. Since this compound has been tested back since 2005 and recently has completed phase III clinical trials and announced for the treatment against intermediate and high-risk patients of myelodysplastic syndromes [207]. Details of other modified nucleotide approach and peptide nucleic acid (PNA) approach targeted against telomerase are available in the literature [210,211,212]. Various approaches regarding the development of effective telomerase immunotherapy have led to the development of useful products. The most advanced products along this line are GRNVAC1 and GV1001 (telomerase peptide vaccine). Both peptide vaccines were particularly designed to enhance an immune response to cancer cell but their efficacy in patients has not been evaluated completely [160,161]. Thus, there is an urgent need to complete evaluation of these compounds to use in combination or alone for certain types of cancer [209].

The strategies listed above offered their prospective advantages and disadvantages. The merit of targeting telomerase is the high specificity for cancer cells due to its high occurrence. Considering as a bearable risk of targeting of normal telomerase positive somatic cells, the prospect to pursue a broad-spectrum cancer therapy propel the scientific researcher over the past twenty years. Somatic cells that express telomerase, such as hematopoietic cells, greatly affected by targeting telomerase activity in progenitor cells as normal hematopoietic stem cells should be less affected by telomerase inhibition therapy than cancerous cells, which almost always have a higher rate of proliferation and thus have higher amount of telomerase activity [213]. 

Telomerase targeting offers two major apprehensions. The first is the time required for telomerase inhibition. Several studies in cultured cells show that effective inhibition of telomerase requiring several weeks to months to stop the proliferation of cells [214]. The second issue raised is resistance mechanisms to telomerase inhibition which lead to progressive shortening of telomere and when telomeres of cancer cells attain a critical length, a crisis phase occur, where strong selective pressure probably favor the growth of resistant cells such as those which start using alternative mechanism of telomere maintenance such as ALT [215,216,217].

### 5.2. Telomeres

The restrictions and prospective challenges to target telomerase enzyme while knowing the fact that inhibition of telomerase would not able to target the one-sixth of melanoma which uses ALT pathway. These challenges further propel researchers to explore telomeres as a healing target [218]. Folding of telomeric DNA into G4 structure blocks the action of telomerase through locking the telomeric single-stranded region into an inactive conformation which is no more available to recognized nor elongated by telomerase [219]. This incapability of telomerase to reach the telomere can activate telomere length-independent damage signal causing instant cell arrest or death. Formation of G4 structure not only affects the function of telomerase but it also inhibits the binding of component of both complexes (shelterin and CST) with single-stranded telomeric DNA [220,221]. For the complete replication of chromosomal ends, it requires unwinding of higher-order G4 structures carried out by a number of helicases such as BLM, WRN, and RecQ along with POT1 [222]. In normal condition, G4 function as a capping structure and its stabilization through G4 ligands block the unwinding which ultimately affects the length of telomere [223].

The G4 structures emerge as a novel therapeutic target to repress telomerase-mediated telomere regulation. The chemical compounds which can block interactions between telomerase and telomere, include G4 ligands that stabilize the G4 structures [224]. In vitro and in vivo studies of G4 ligands have shown to have a promising anticancer activity which leads to the search for small molecules that specifically interact and stabilize the G4 structure [225,226]. There are two key limitations on the use of G4 stabilizing molecules are, one is the lack of potency and second is poor selectivity between G4 and DNA duplex [227]. Telomestatin is a widely studied inhibitors reported to promote telomere shortening and to block proliferation of cancer cells in vitro and in model organisms [228]. In 2013, the first NMR structure of G4 in complex with Telomestatin was solved by Wan Jun Chung and co-worker [229]. This observation pushes forward researcher for the design and synthesis of topology specific G4-targeting compounds valuable for the development of effective anticancer drugs [229].

In consistence, RHPS4 is a pentacyclic acridinium ligand form complex with G4 through end- stacking mechanism. The π-system of RHPS4 overlaps mainly with two bases of each tetrad through stacking interactions with the G-tetrads [230,231]. Further studies show that the RHPS4 abrogates the function of telomerase and subsequently promotes telomere uncapping and finally leads to senescence [167]. The crystal structure of a 3,6,9-trisubstituted acridine, BRACO-19, in complex with G4 were solved by Campbell et al. [232]. BRACO-19 is sandwiched between two quadruplexes to form a biological unit in a way such that the G4 are uniquely stacked 5′–3′ direction. Moreover, a number of modifications on BRACO-19 have been made and those modified molecules shows strong binding affinity with G4 along with telomerase inhibitory activity and exhibits anti-proliferation property on cancer cell lines [166,192,233]. There are number of other important G4 ligands such as quinacridine analogs (MMQ1), quarfloxin, naphthalene diimides (ND), porphyrin analogue (TMPyP4) that show a strong binding with G4 together with anti-cancer activity [169,234,235,236,237]. In addition to targeting both components of telomerase and G4 structures, targeting components of shelterin and CST complexes is also useful in the treatment of telomeropathies.

Compounds targeting the telomere are actually obstructs its stability. Telomeres are found in normal as well in cancerous cells, and therefore the risk of cytotoxicity in the track of such approaches is concrete. Reports on the effects of telomere targeting in normal cells are existing mainly for G4 ligands which reveal a higher resistance in normal cells compared to cancer cells [167,238,239]. Another concern raised by the telomeres targeting by G4 ligands is that even the most specific telomeric G4 ligand retains the ability to bind other G4 structure in regulatory regions or other gene promoters of the genome [240,241]. 

### 5.3. Shelterin Components

Targeting shelterin components for cancer therapy has emerged a few years ago. POT1 is targeted by a berberine derivative (Sysu-00692) which disrupts its interaction POTI with telomeric DNA [77]. Another report suggested that TRF2 as a potential therapeutic target of cancer. A well-known anti-cancerous compound, gemcitabine, a nucleoside analog approved as anti-cancer agent [156,174,242]. Since gemcitabine is a nucleoside analog which acts via incorporating into DNA in place of cytosine and consequently inhibits DNA replication and promotes telomere loss through TRF2 stabilization [156]. Further studies reveal that a metronomic treatment with gemcitabine has anti-angiogenic effects in a pancreatic cancer model [173,243]. However, care must be taken when using drugs molecule targeting components of shelterin in anti-cancer therapy as the shelterin complex is crucial for monitoring telomere stability in normal cells as well. 

Recently, two novel compounds (ETP-47228 and ETP-47037) have been identified which abrogate the function of one of the important telomeres uncapping protein, TRF1. This abrogation effectively hampers the growth of previously established lung carcinomas without disturbing tissue viability [175]. From this study, targeting of shelterin components got strong support and provides proof of the concept that abrogation of shelterin component could be a useful therapeutic approach to inhibit the growth of various types of carcinomas.

Another strategy to target telomere-associated disease is to manipulate the t-loop, which forms by the invasion of the telomere single-stranded 3′ G-overhang into the duplex chromosomal end through nucleotide base pairing [6]. One of the shelterin components, TRF2 is sufficient and makes the process easy to form t-loop [244,245]. According to three state models (hypothetical), it assumed that t-loop formation hides telomeric end to recognize as double-stranded breaks [155]. Loss of t-loop formation resulted int the activation of ATM, p53 and many other downstream molecules which finally form TIF (telomere-dysfunction induced foci) and initiate many growth inhibitory responses including senescence, cell cycle arrest, or apoptosis [246]. In addition, it has been demonstrated that the use of oligonucleotide or t-oligos having homology to the G-rich region of t-loop able to induce apoptosis, senescence, or autophagy in a range of melanoma consistent with disruption of telomere loop [177,179,247,248,249]. Even though much work has been done but still there is a strong need to understand and explore the mechanism of t-loops in chromosomal end protection in a model system.

Another but the unexplored approach in targeting telomere binding proteins subject to a range of post-translational modification (PTM) including phosphorylation, ubiquitylation, SUMOylation, PARsylation. These PTM carried out by different types of kinases such as CK2, Cdkl, Fbx4, and MMS21 Tankyrase-1 and 2), which eventually control and regulate the activity and function of telomere binding protein. The above four types of PTM (phosphorylation, ubiquitylation, SUMOylation, and PARsylation) have been found on TRF1 and TRF2, except methylation which primarily found on TRF2 protein. These modifications of TRF1 and TRF2 regulate key aspects of TRF1 and TRF2 function at the chromosomal ends such as stability of TRF1 or TRF2, telomeric DNA binding, protein–protein interactions and priming of TRF1 for subsequent modification [33,250].

**Figure 6:**
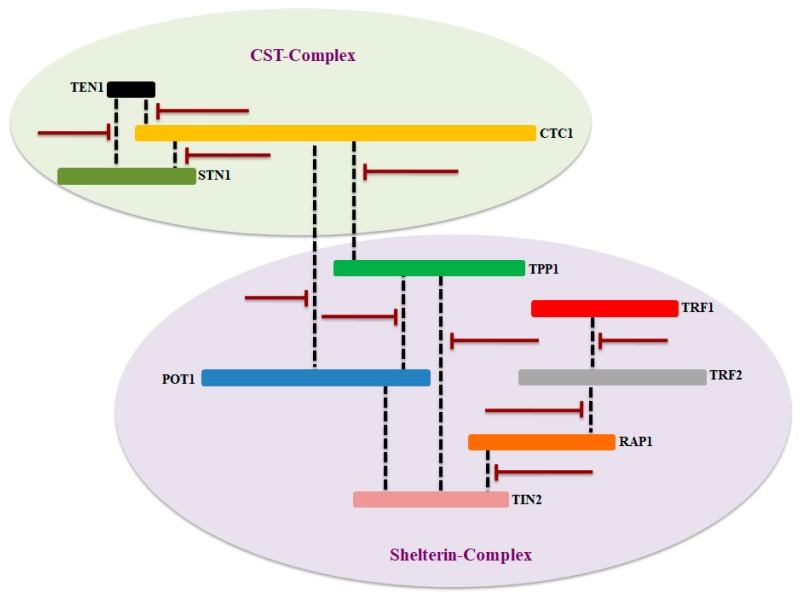
Proposed key target site in CST and shelterin complex that can be targeted for therapeutic applications. Some of the key interacting partners of these complexes have been considering as an emerging targets for telomere borne diseases, while others are being investigation for therapeutic targets. The dashes line in black indicates the interaction between components of both complexes and bar-headed lines showing possible inhibition site in red.

Interestingly, TANK1 and 2 modifies TRF1 and start the release of TRF1 from telomere which has emerged as a therapeutic target for cancer [181,251,252]. It was demonstrated that acetylation of TRF2 through p300 control TRF2 stability and thus telomere binding. High expression of TRF2 mutant (lacking acetyl modification) promotes distorted telomeres, telomeric DDR and senescence [253]. Similarly, TPP1 also shows a strong link between PTM of TPP1 and telomere regulation [254]. As small molecule inhibitors of many key enzymes which catalyzes these modifications are available commercially. In-depth understanding of these modifications control and regulate telomere protection and synthesis which may help the researcher to change the telomere status and immortalized cancer cells through inhibiting the crucial enzymes important for PTM of telomere binding proteins. Although, the CST complex facilitates the replication of telomeric DNA, mediate C-strand fill-in, and inhibit unnecessary elongation of G-strand [83].Currently, there are no cancer therapies available that exclusively target CST complex. To come up with novel possible ways to target these complexes, some hypothetical approaches are outlined in Figure 6.

Telomerase/telomere or inhibitors of telomere maintaining proteins might not only directly limit the growth of the cancerous cells, but could become more effective using in a synergistic fashion with existing therapeutic modalities such as with chemotherapeutic agents and anti-angiogenic agents [213,255,256].

## 6. Conclusions

To identify potential drug targets, knowledge from structures, biochemical features and mechanistic studies of telomerase, CST and shelterin complexes must be comprehensively employed to evaluate potential targets for translational setting against telomere borne diseases. After extensive analysis of each component of both complexes, we have identified approximately 115 clinically important mutations. Most of these mutations either affect the telomere protection or its replication process. Majority of the mutations in both complexes are implicated in several diseases including cancer, idiopathic pulmonary fibrosis, DC, CP bone marrow failure and premature aging syndromes. In addition, identification of mutations in both complexes and its association with disease raises the question that how mutation in these genes does affect the pathogenesis of different disease? Targeting telomerase with specific telomere structure (G4) to inhibit its function is a conventional strategy to fight cancer and other associated diseases. However, a great lacuna of potential drug is essentially required to address the components of CST and shelterin complexes in broader aspects. Taken together, we conclude that the emergence of innovative drug development strategies that addresses novel targets could provide a roadmap for the development of potential inhibitors with the capacity for simultaneous disruption of multiple tumor cell dependencies including those promoted by telomerase and telomere regulatory proteins (CST and shelterin complex). In addition, targeting protein–protein interaction could be useful to interrupt the interaction between components of these complexes. 

## Figures and Tables

**Table 1:** Some clinically important mutations, associated genes, and their corresponding cellular pathologies. Among both complexes, protection of telomeres 1 (POT1) and conserved telomere maintenance component 1 (CTC1) appear as most recurrent mutated genes.

Gene	Mutation	Type of Disease	Reference
*RAP1*	p.M5I	Familial Melanoma	[43,44]
	p.D10H		
	p.Q191R		
	p.R364X		
	p.A104P	Chronic lymphocytic leukemia	
	p.R133Q		
*TIN2*	p.R282H	Dyskeratosis congenita	[55,89,90,91,92,93]
	p.R282S		
	p.K280E		
	p.R282C		
	p.L280X		
	p.R282H		
	p.R282S		
	p.R282L		
	p.Q269X		
	p.Q271X		
	p.P283S		
	p.P283A		
	p.P283H		
	p.T284A		
	p.T284H		
	p.L287P		
	p.P289S		
	p.F290LfsX2		
	p.R291G		
	p.Q298R fsX19		
	p.K280RfsX36		
	p.A43T	N/A	
	p.G237D		
	p.P241S		
	p.P236S		
	p.E281K		
	p.S245Y	Pulmonary fibrosis	[56]
*TPP1*	p.G223V	Acute lymphoblastic leukemia	[94]
	p.K170X	Dyskeratosis congenita	[95]
	p.P491T		
	p.R159A;E160A	N/A	[64]
	p.D163A;T164A		
	p.D166R;E168R		
	p.S111A		
*POT1*	p.Y89C	Familial melanoma	[96]
	p.Q94E		
	p.R273L		
	p.R137H	Cutaneous malignant melanoma	[97]
	p.D224N		
	p.S270N		
	p.A532P		
	p.Q623H		
	p.G95C	Familial glioma	[98]
	p.E450X		
	p.D617E		
*POT1*	p.R117C	Cardiac angiosarcoma	[99]
	p.S322L	Coat plus	[100]
	p.M1L	Chronic lymphocytic leukemia	[44,101,102]
	p.Y36N		
	p.Y66X		
	p.K90Q		
	p.Q94R		
	p.Y223C		
	p.S250X		
	p.H266L		
	p.G272V		
	p.C591W		
	p.Y36C		
	p.Q376R		
	p.Q358S		
*CTC1*	p.A227V	Coat plus	[94,103,104,105,106]
	p.R975G		
	p.R840W		
	p.R987W		
	p.G503R		
	p.L1142H		
	p.V665G		
	p.V259M		
	p.V871M		
	p.R1196X		
	p.R287X		
	p.R840W		
	p.L1142H		
	p.V665G		
	p.V259M		
	p.V871M		
	p.H484P		
	p.G278V		
	p.Y281H		
	p.K242L	Dyskeratosis congenita	[107,108]
	p.C985X		
	p.L464V		
	p.L247P		
	p.G278V		
	p.C424G		
	p.I1005V	N/A	[106]
	p.H484H		
	p.I820V		
*STN1*	p.R135T	Coat plus	[84]
	p.D157Y		
	p.W89A	N/A	[82,109]
	p.R139L		
	p.Y141A		
	p.S248C		
	p.T151A		
	p.D78A		
	p.I164A		
*TEN1*	p.R27Q	N/A	[82]
	p.Y115A		
	p.R119Q		

N/A = Not available.

**Table 2:** List of important small molecule inhibitors and their mechanism of action.

Target	Mechanism	Compound	Reference
Telomerase	Target TERT	BIBR1532	[141,142,143,144,145,146,147]
		TMPI	[148]
		EGCG and MST312	[149,150]
		AZT	[151,152,153]
	Target hTR	Imetelstat	[77,82,154,155,156,157,158,159]
	Peptide vaccine	GRNVAC1, GV1001	[160,161]
Telomere	Target G-quadruplex	Telomestatin	[162,163,164]
		RHPS4	[165,166,167,168]
		BRACO-19	
		Quarfloxin	[169,170]
		TMPyP4	[171,172]
CST complex	CTC1, STN1, TEN1	N/A	N/A
Shelterin	Target POT1	SYSU-00692	[77]
	Target TFR2	Gemcitabine	[156,173,174]
	Target TFR1	ETP-47228, ETP-47037	[175]
T-loop	Target t-loop	t-oligos	[176,177,178,179,180]
Post-translational modifications	TRF1-PARsylation	PARP inhibitors	[181,182,183]
